# 3D Binder-Free Mo@CoO Electrodes Directly Manufactured in One Step via Electric Discharge Machining for In-Plane Microsupercapacitor Application

**DOI:** 10.3390/mi15111294

**Published:** 2024-10-24

**Authors:** Shunqi Yang, Ri Chen, Fu Huang, Wenxia Wang, Igor Zhitomirsky

**Affiliations:** 1 College of Informatics, Huazhong Agricultural University, Wuhan 430070, China; yyssqqq@webmail.hzau.edu.cn; 2 Department of Mechatronic Engineering, Guangdong Polytechnic Normal University, Guangzhou 510665, China; 3 Department of Biomedical and Pharmaceutical Sciences, Guangdong University of Technology, Guangzhou 510006, China; fewwxia@gdut.edu.cn; 4 School of Materials Science and Engineering, McMaster University, Hamilton, ON L8S 4L7, Canada; zhitom@mcmaster.ca

**Keywords:** cobalt oxide, in-plane microsupercapacitors, electric discharging machining, integrated microelectrodes, Mo@Co-CoO

## Abstract

Cobalt oxide-based in-plane microsupercapacitors (IPMSCs) stand out as a favorable choice for various applications in energy sources for the Internet of Things (IoT) and other microelectronic devices due to their abundant natural resources and high theoretical specific capacitance. However, the low electronic conductivity of cobalt oxide greatly hinders its further application in energy storage devices. Herein, a new manufacturing method of electric discharging machining (EDM), which is simple, safe, efficient, and environment-friendly, has been developed for synthesizing Mo-doped and oxygen-vacancy-enriched Co-CoO (Mo@Co-CoO) integrated microelectrodes for efficiently constructing Mo@Co-CoO IPMSCs with customized structures in a single step for the first time. The Mo@Co-CoO IPMSCs with three loops (IPMSCs3) exhibited a maximum areal capacitance of 30.4 mF cm^−2^ at 2 mV s^−1^. Moreover, the Mo@Co-CoO IPMSCs3 showed good capacitive behavior at a super-high scanning rate of 100 V s^−1^, which is around 500–1000 times higher than most reported CoO-based electrodes. It is important to note that the IPMSCs were fabricated using a one-step EDM process without any assistance of other material processing techniques, toxic chemicals, low conductivity binders, exceptional current collectors, and conductive fillers. This novel fabrication method developed in this research opens a new avenue to simplify material synthesis, providing a novel way for realizing intelligent, digital, and green manufacturing of various metal oxide materials, microelectrodes, and microdevices.

## 1. Introduction

In recent years, the Internet of Things (IoT) has led to the broad application of small-size devices, which have become an integral part of daily life [1,2,3,4,5]. IoT enables the connection of sensors and devices through networks to facilitate data sharing and exchange. It encompasses a wide range of fields, including smart home technology, industrial automation, healthcare, transportation, agriculture, and more. By interconnecting devices, IoT offers society more efficient, intelligent, and convenient services [6,7,8,9]. Typically, these sensors should transmit data both remotely and periodically to achieve a sustainable and long-lasting autonomous power supply. Triboelectric nanogenerators, solar cells, and wind power are widely used to power these devices. However, as these periodic energy sources are not able to store energy themselves, they require an additional unit for storing energy [10,11,12]. Lithium-ion batteries seem to be a good option due to their high specific energy density. Unfortunately, the cycle life of lithium-ion batteries has not yet been significantly extended by modern technology, resulting in frequent replacement of lithium-ion batteries and high maintenance costs. Moreover, the limited natural source of lithium on Earth furtherly restricts its broad applications [13,14,15,16]. In contrast, microsupercapacitors (MSCs) have garnered broad interest from researchers because of their satisfactory advantages of low operation costs, high safety, fast charging/discharging rate, long operating life, and high-specific energy density. The electrode materials utilized in the fabrication of supercapacitors could be classified into three separate categories based on their energy storage mechanisms [5,17,18]: (1) The predominant class is carbon-related materials utilizing the electric double-layer capacitance mechanism for energy storage. (2) A fast Faraday redox reaction is involved in the energy storage mechanism of conducting polymers (like polypyrrole (PPy) and poly(3,4-ethylene dioxythiophene) (PEDOT)) and transitional metal hydroxides/oxides. (3) Composite materials display characteristics of both electrostatic double-layer capacitance and redox reactions. Currently, the development of MSCs is limited by their low energy density. Researchers have made significant efforts to raise the specific energy density of MSCs through the synthesis of advanced composite materials, nanostructured materials, foreign element doping materials, and vacancy-enriched materials [19,20,21,22]. Among the various electrode materials, transition metal oxides with relatively high theoretical capacitance and good cyclic stability have attracted lots of attention from researchers [23,24,25]. For instance, RuO_2_ is a transition metal oxide known for its high electrochemical activity, high electronic conductivity, high theoretical capacitance, and excellent cycling stability [26,27,28]. However, the high cost, limited natural reserves, and toxic properties of RuO_2_ greatly hinder its broad application in MSCs. As a result, research has sought alternative materials with similar properties. Recently, a number of alternative metal oxides have been developed, including MnO_2_ [29,30], MoO_x_ [31], VO_0.2_ [32], FeO_x_ [33,34,35,36], CoO [37], ZnO [38], and NiO [39,40]. Among these metal oxides, CoO is one of the highly promising materials for MSCs as it demonstrates excellent chemical stability and high capacitance in theory [37]. Nevertheless, they encounter certain obstacles in their practical applications, such as poor electronic conductivity of CoO. To address these challenges, researchers are investigating various approaches, such as designing nanostructured materials, integrating CoO with high-electronic conductivity materials (such as graphene and carbon nanotube) to form composite materials, introducing foreign element doping, or creating vacancy-enriched materials [17,41,42,43]. For example, Zhou et al. [44] synthesized CoO with well-aligned nanowire nanostructure on three-dimensional Nickel foam coated with polypyrrole (PPy) to achieve CoO/PPy composite electrodes. Benefiting from the synergistic effect of nanostructured CoO and the high electronic conductivity of PPy, these CoO/PPy composite electrodes obtained an excellent capacitance of 2223 F g^−1^ with good rate performance and remarkable capacitance retention of 99.8% (2000 cycles). Gao et al. [45] also prepared graphene/Co/CoO nanocomposite fork-finger electrodes via laser direct writing. These electrodes showed a capacitance of 420.23 mF cm^−2^ with superior capacitance retention of 86.56% after 10,000 cycles. These results demonstrate that MSCs fabricated with CoO-based composite electrodes possess remarkable specific capacitance and impressive cycling stability. Liu et al. [25] synthesized a novel Cu^0^/Cu^+^-codoped CoO composite, which demonstrated a notable strengthening in the capacitive performance of the CoO electrode, as evidenced by the observed enhancement in the capacitance and cycling stability. This improvement is attributed to the introduction of metal element doping, which greatly increased the electronic conductivity of CoO materials. Recently, Feng et al. [46] prepared CoO-based electrode materials with dual defects—Cu doping and oxygen vacancies. The results demonstrated that the CoO-based electrode gained a remarkably high capacitance value of 1389 F g^−1^ at 1 A g^−1^ and good capacitance retention of 101% (10,000 cycles). The enhanced capacitive performance of the CoO-based electrode is attributed to the introduction of Cu doping and oxygen vacancies, which greatly improved the electronic conductivity of CoO. Previous studies have shown that metal ion doping and oxygen vacancies can modulate the structure and chemical reactivity of CoO, resulting in materials with excellent electrochemical properties. Although the aforementioned methods could enhance the electrochemical performance of CoO electrodes through metal ion doping and oxygen vacancies, the fabrication steps are overly complex, often involve toxic chemicals, and require specific synthetic environments, which greatly limit the potential for their widespread application. In addition, up to now, there has been no report on the synthesis of CoO with dual defects—oxygen vacancy and metal doping—for IPMSC application. Therefore, there is a pressing necessity for the development of a streamlined and expeditious manufacturing process to create high-performance CoO-based microelectrodes with dual defects (oxygen vacancy and Mo doping) through a single-step approach for IPMSC applications. It has been reported that electric discharging machining (EDM) is a non-contact machining strategy triggered by an applied voltage across a narrow gap between the machining tool and the machining part. This EDM technique allows for the machining of conductive substrates regardless of their stiffness and strength and is used in many applications, such as aviation, electronics, and automation [47,48]. Benefiting from the numerically controlled systems, EDM facilitates machining workpieces with different geometric patterns in a single step. Moreover, it has been proved that cobalt oxide can be synthesized through a one-step EDM process [49]. However, up to now, there is no report on the investigation of the electrochemical performance of cobalt oxide generated via EDM for supercapacitor applications.

Herein, a convenient, simple, and environmentally friendly computer-aided EDM processing method has been developed for synthesizing Mo-doped and oxygen-vacancy-enriched Co-CoO–integrated microelectrodes without the utilization of extra conductive agents, adhesives, and current collectors. This method efficiently constructs Mo@Co-CoO IPMSCs with a customized structure in a single step. The Mo@Co-CoO IPMSCs3 achieved a maximum capacitance of 30.4 mF cm^−2^ at 2 mV s^−1^. Moreover, the Mo@Co-CoO IPMSCs3 showed good capacitive behavior at a super-high scanning rate of 100 V s^−1^, which is around 500–1000 times higher than those reported for CoO-based electrodes, which normally operate within 100–200 mV s^−1^. This remarkable performance is attributed to the dual defects of oxygen vacancies and Mo doping, along with the 3D binder-free integrated electrode design, which significantly boosts the electrochemical performance of CoO-based microelectrodes. It is important to note that the Mo@Co-CoO IPMSCs were fabricated using one-step EDM without any assistance of other material processing techniques, toxic chemicals, low conductivity binders, exceptional current collectors, and conductive fillers. This fabrication method developed in this research opens a new avenue for simplifying the material synthesis procedures and enables the one-step synthesis of various metal oxide materials with metal ion doping and oxygen vacancies for different applications, such as IPMSCs, batteries, and catalysts.

## 2. Material Sources and Experiments

### 2.1. Material Sources

The cobalt metal substrate was obtained from a company named Qing He Lisheng Metal Materials (Xingtai, China). The molybdenum wire for electric discharge machining was purchased from Jin Duicheng Molybdenum Mining of Guang Ming (Zibo City, China). The KOH solution with a concentration of 1 M was obtained from Kell Chemical Technology (Guangzhou, China).

### 2.2. Manufacturing of Mo@Co-CoO–Based Microelectrodes for IPMSCs

Mo@Co-CoO–based integrative electrodes were achieved by numerically machining the cobalt metal substrate via the EDM technique. Thereafter, EDM was used to machine these integrated electrodes into 3D Mo@Co-CoO–based IPMSCs with various geometries, such as IPMSCs with one loop (named IPMSCs1), IPMSCs with two loops (named IPMSCs2), and IPMSCs with three loops (named IPMSCs3). Moreover, IPMSCs1 before EDM treatment (named IPMSCsB1) was assembled for comparison. For IPMSCsB1, the surface of the Co substrate was polished but not treated with EDM.

### 2.3. Characterization for Materials and IPMSC Devices

The analysis of electrode surface morphology was conducted using scanning electron microscopy (SEM) from the TESCAN brand (Brno, Czech Republic). The element distribution of the Mo@Co-CoO electrode was investigated with Energy dispersive spectroscopy (EDS) using a TESCAN MIRA LMS system. The composition and valence state of the electrode materials were examined using X-ray photoelectron spectroscopy (XPS) with a Thermo Scientific K-Alpha instrument. X-ray diffraction (XRD) analysis was performed with a Rigaku CuKα diffractometer to investigate the composition of the Mo@Co-CoO–integrated electrode. Electron Paramagnetic Resonance (EPR) was carried out to prove the oxygen vacancies in the Mo@Co-CoO electrode using a Bruker EMXplus–6/1 system. Raman spectra were collected using a Horiba LabRAM HR Evolution spectrometer with a 532 nm excitation laser. The CHI660E, purchased from Chenhua Shanghai, China, was utilized for the electrochemical characterization of cyclic voltammetry (CV) and galvanostatic charge–discharge (GCD). The CV examination was carried out from 2 to 100,000 mV s^−1^ within 0–0.6 V, whereas the GCD investigation was conducted from 0.2 to 1 mA cm^−2^ at the same voltage window. The KOH solution with a concentration of 1 M was utilized as the electrolyte for electrochemical characterization.

## 3. Results and Discussion

**Figure 1:**
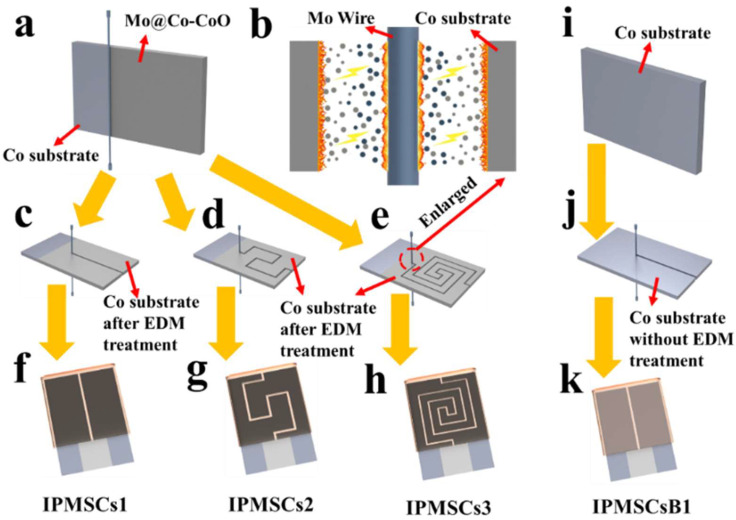
Schematic images for manufacturing IPMSCs: (**a**) machining process and (**b**) machining mechanism of the Co substrate via EDM; (**c**–**e**) machining of Mo@Co-CoO integrated electrodes into designed patterns; (**f**–**h**) schematic diagram of the fabricated microdevices IPMSCs1, IPMSCs2, and IPMSCs3; (**i**–**k**) schematic fabrication process of IPMSCsB1.

Figure 1 demonstrates the simple machining procedures for Mo@Co-CoO electrodes and microdevices utilizing a Co metal substrate as the starting material. The Co metal substrate not only acts as a current collector for the devices but also is used as the main material source for synthesizing CoO-based active materials for IPMSC applications. During the EDM process, sparks generated between the Co metal substrate and the Mo metal wire result in an ultrahigh temperature in the discharge channel, leading to the melting of the Co metal substrate and the Mo metal wire (Figure 1b). When the EDM processing was turned off, these melting materials were rapidly cooled down by the flowing deionized water and solidified on the surface of the Co current collector, which finally formed binder-free integrated Mo@Co-CoO electrodes for IPMSCs. Thereafter, these binder-free Mo@Co-CoO electrodes were machined into various IPMSCs with customized patterns, designated as IPMSCs1, IPMSCs2, and IPMSCs2 (Figure 1c–h). In comparison, the surface of the Co substrate without EDM treatment was shaped into IPMSCsB1 with one loop (Figure 1i–k).

**Figure 2:**
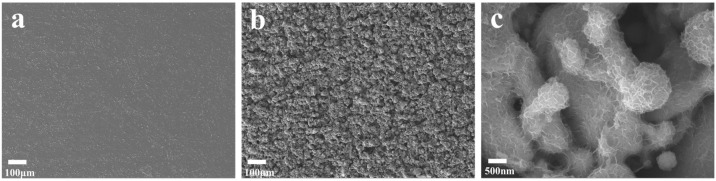
SEM images of (**a**) the Co substrate before EDM processing and (**b**,**c**) Mo@C-CoO electrodes at (**b**) low and (**c**) high magnifications.

**Figure 3:**
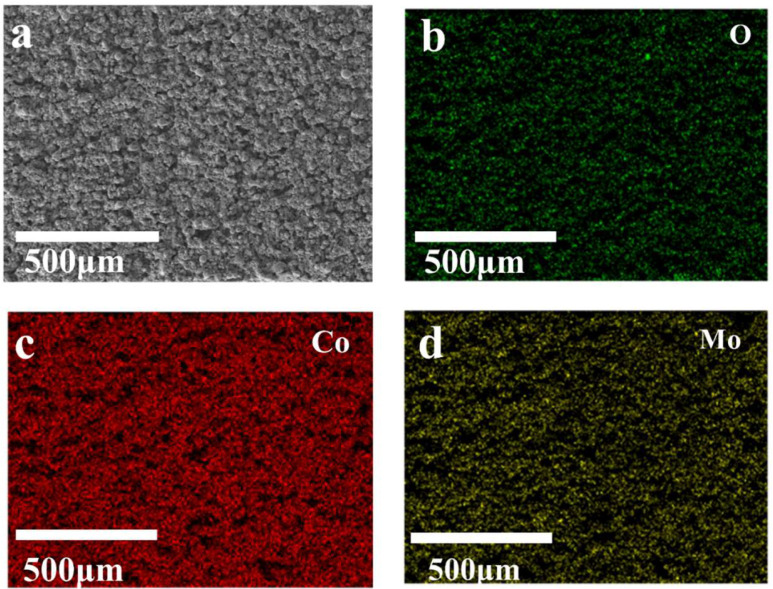
(**a**) SEM images of Mo@C-CoO electrodes after EDM processing and (**b**–**d**) elemental distribution of O, Co, and Mo on the surface of Mo@C-CoO electrodes, as proved by EDS analysis.

**Figure 4:**
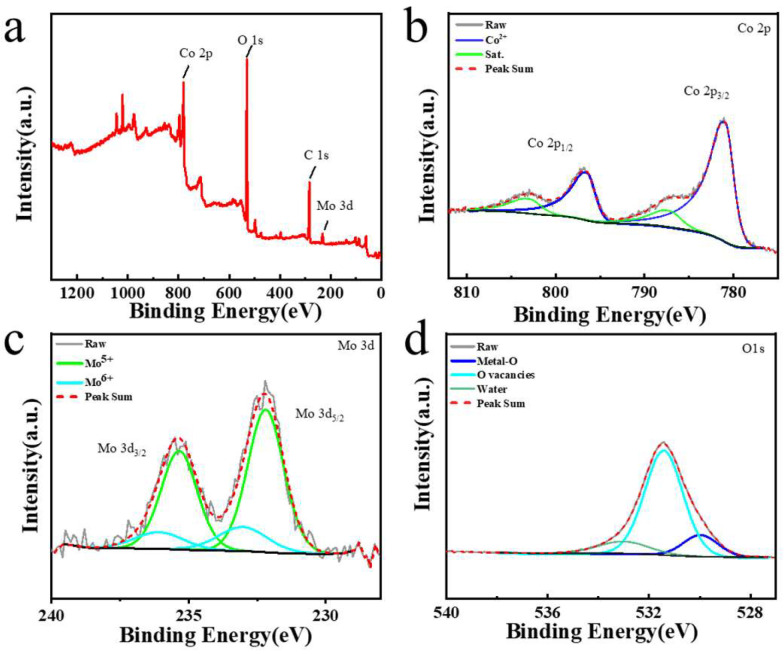
XPS examination curves of Mo@Co-CoO electrodes: (**a**) survey scan, (**b**) Co 2p, (**c**) Mo 3d, and (**d**) O 1s.

SEM examination was carried out to investigate the surface characteristics of the Co metal substrate without and with EDM processing. The results show that the Co substrate without EDM processing is very smooth (Figure 2a), whereas the Co substrate machined using EDM becomes rough and grows plenty of microstructures enriched with nanostructures (Figure 2b,c), offering lots of active sites for ion adsorption/desorption. Elemental distribution analysis of the three elements—Mo, Co, and O—on the surface of the machining electrodes was performed using EDS examination (Figure 3). It was found that all these three elements, Mo, Co, and O, are uniformly distributed on the electrode, confirming that the EDM strategy facilitates the synthesis of Mo@Co-CoO–integrated electrodes with good surface quality. XPS study was used to further investigate the composition of the Mo@Co-CoO–integrated electrodes (Figure 4). All the XPS spectra were standardized by referencing the C1s peak at 284.8 eV. As shown in Figure 4a, the XPS full survey spectrum confirms the presence of Co, Mo, O, and C as the predominant elements in the Mo@Co-CoO electrode. The fitting results of the Co 2p and Mo 3d spectra are displayed in Figure 4b and Figure 4c, respectively. In Figure 4b, two characteristic peaks observed at 780.8 eV and 796.5 eV can be assigned to Co 2p_3/2_ and Co 2p_1/2_ of the standard CoO composition, while the satellite peaks at 787.1 eV and 802.9 eV further confirm the formation of CoO [50,51]. In Figure 4c, the peaks at 233.0 eV and 236.1 eV stand for the Mo^6+^ 3d_5/2_ and Mo^6+^ 3d_3/2_ orbitals, while the peaks at 232.2 eV and 235.4 eV can be assigned to the Mo^5+^ 3d_5/2_ and Mo^5+^ 3d_3/2_ orbitals, confirming the successful incorporation of Mo into the structure [51,52]. The O 1s spectrum (Figure 4d) was deconvoluted into three peaks at 530.0, 531.5, and 533.0 eV, representing metal–O bonds, oxygen vacancies, and surface-adsorbed water, respectively [53,54]. Figure S1 presents the Raman spectrum of the prepared Mo@Co-CoO electrode, indicating that the prominent Raman bands at 310 cm^−1^ and 829 cm^−1^ stand for the bending and stretching modes of Mo=O, respectively. The band at 660 cm^−1^ is attributed to the symmetric stretching mode of Mo-O-Mo [51,55]. Moreover, the band at 526 cm^−1^ is associated with the Co-O vibration mode [56]. These Raman results further confirmed the Mo doping in the CoO electrode. In addition, the generation of oxygen vacancies was further proved by EPR testing (Figure S2). The Mo@Co-CoO samples gained a g-value of 2.0058, which is close to the free electron value of 2.0023. This EPR result proved that unpaired electrons were successfully introduced into Mo@Co-CoO [46,57,58]. The presence of Mo doping and oxygen vacancies accelerates the electronic/ionic transfer rates of the Co-CoO–integrated electrodes, thus boosting their capacitive performance. Moreover, XRD was used to further reveal the chemical microscopic composition of the Mo@Co-CoO electrode. In Figure S3, three prominent peaks were observed at 2θ values of 43.9°, 51.2°, and 75.2°, corresponding to the (111), (002), and (022) planes of Co (JCPDS card 01-2927), respectively [59]. Additionally, weak peaks at 35.3°, 41.5°, and 60.2° represent the (111), (200), and (220) planes of CoO (JCPDS card 43-1004) [49,51].

**Figure 5:**
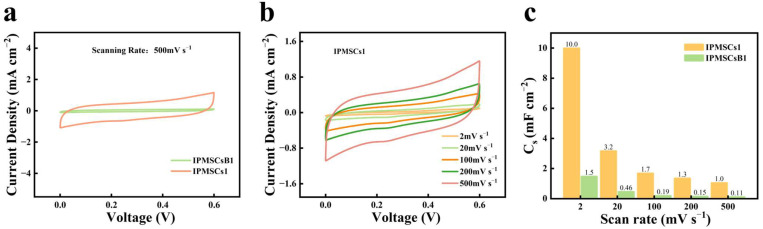
(**a**) CV profiles of IPMSCsB1 without EDM treatment and IPMSCs1 after EDM at 500 mV s^−1^; (**b**) CV profiles of IPMSCs1 at scanning rates of 2–500 mV s^−1^; (**c**) areal capacitance of IPMSCsB1 and IPMSCs1.

**Figure 6:**
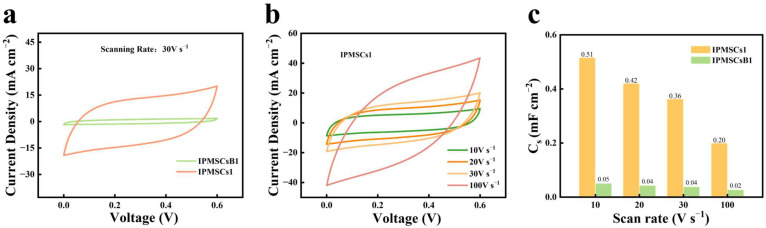
(**a**) CV profiles of IPMSCsB1 without EDM treatment and IPMSCs1 after EDM at ultrahigh testing condition of 30 V s^−1^; (**b**) CV profiles of IPMSCs1 at testing conditions of 10–100 V s^−1^; (**c**) areal capacitance of IPMSCsB1 and IPMSCs1.

The CV studies of 3D binder-free IPMSC microdevices with various geometric shapes (IPMSCs1, IPMSCs2, and IPMSCs3) machined via EDM and the reference sample (IPMSCsB1) without EDM treatment were conducted at scanning rates of 2–100,000 mV s^−1^. Figure 5a shows the CV profiles of IPMSCs1 and IPMSCsB1 at 500 mV s^−1^. The results demonstrate that the IPMSCs1 microdevice, machined via EDM, illustrated a much larger current density than that of IPMSCsB1 without EDM treatment. This is attributed to the synthesis of Mo@Co-CoO microelectrode materials, which are equipped with plenty of micro and nanostructures, facilitating efficient active sites for ion adsorption and desorption. The CV curves of the IPMSCs1 device at varying scanning rates are shown in Figure 5b. All CV profiles display a near box shape, which proved that the IPMSCs1 device achieves good capacitive performance. Figure 5c further indicates that the IPMSCs1 microdevice exhibits a much larger areal capacitance than that of IPMSCsB1. Moreover, comparative studies between IPMSCs1 and IPMSCsB1 were conducted under super-high charging/discharging conditions of 10, 20, 30, and 100 V s^−1^ (Figure 6). The results prove that the capacitance of IPMSCs1 is almost 10 times higher than that of IPMSCsB1. This observed phenomenon agrees well with the results presented in Figure 5.

**Figure 7:**
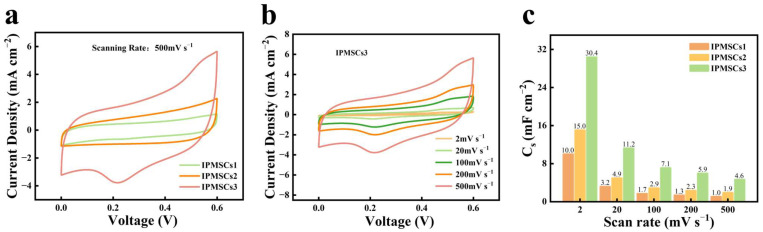
(**a**) CV profiles of IPMSCs1, IPMSCs2, and IPMSCs3 manufactured via EDM at 500 mV s^−1^; (**b**) CV profiles of IPMSCs3 at testing parameters of 2–500 mV s^−1^; (**c**) related capacitance of IPMSCs1, IPMSCs2, and IPMSCs3.

**Figure 8:**
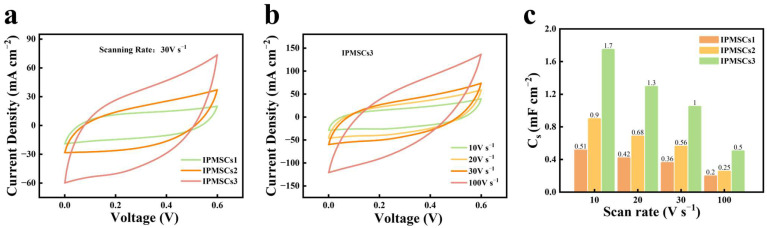
(**a**) CV profiles of IPMSCs1, IPMSCs2, and IPMSCs3 manufactured via EDM at ultrhigh testing condition of 30 V s^−1^; (**b**) CV profiles of IPMSCs3 at testing conditions of 10–100 V s^−1^; (**c**) related capacitance of IPMSCs1, IPMSCs2, and IPMSCs3.

Moreover, as the EDM machine is equipped with a computer-aided design system, it can efficiently design and fabricate IPMSCs with various geometries. Therefore, the geometric effect on the electrochemical performance of IPMSCs was investigated (Figure 7). Figure 7a shows the CV curves for the microdevices IPMSC1, IPMSC2, and IPMSC3. It can be seen that the IPMSCs3 microdevice exhibits the largest CV area, while the IPMSCs1 microdevice exhibits the smallest one. All the CV curves behave nearly rectangle shapes, indicating that the IPMSC microdevices machined via the EDM method achieve good electrochemical performance. This is attributed to the EDM strategy, facilitating the design of binder-free integrated microelectrode, as well as the introduction of Mo doping and oxygen vacancies, both of which are helpful for accelerate the electronic/ionic transportation rates. Figure 7c illustrates the derived capacitances of IPMSC1, IPMSC2, and IPMSC3 microdevices based on the corresponding CV curves. Among these three microdevices, IPMSC3 achieves the largest areal capacitance of 30.4 mF cm^−2^ (2 mV s^−1^), which is around 3 times and 2 times higher than those of IPMSC1 and IPMSC2, respectively. The same phenomenon was observed for IPMSC1, IPMSC2, and IPMSC3 microdevices at other scanning rates. This enhancement in electrochemical performance is due to the decrease in electrode width, which shortens the ion transport distance, resulting in the enhancement of their electrochemical performance [60,61,62]. Moreover, Table S1 shows that the areal capacitance of IPMSC3 microdevices is higher than that of several reported supercapacitors, such as the V_2_O_5_//PANI-based device (12.3 mF cm^−2^) [63], V_2_O_5_//rGO-based device (24 mF cm^−2^) [64], graphene-PEDOT–based device (5.4 mF cm^−2^) [65], MXene-based device (23 mF cm^−2^) [66], Carbon nanotubes (CNT)-based device (6.1 mF cm^−2^) [67], activated carbon-based device (5.1 mF cm^−2^) [68], carbon onion-based device (1.7 mF cm^−2^) [69], graphene-based device (0.08 mF cm^−2^) [70], another graphene-based device (2.3 mF cm^−2^) [71], and reduced graphene oxide (rGO)-based device (0.51 mF cm^−2^) [72]. Further examination of the capacitive performance of the IPMSCs1, IPMSCs2, and IPMSCs3 microdevices under super-high charging/discharging conditions of 10, 20, 30, and 100 V s^−1^ was carried out and is presented in Figure 8. Figure 8a illustrates the CV profiles of IPMSCs1, IPMSCs2, and IPMSCs3 manufactured via EDM at an ultrahigh testing condition of 30 V s^−1^. It can be observed that the IPMSCs3 microdevice exhibits the largest CV area, while the IPMSCs1 microdevice exhibits the smallest one, which is in good agreement with the results shown in Figure 7a. Furthermore, all the CV curves behaved nearly as rectangle shapes, indicating that the IPMSC microdevices machined via the EDM technique achieve good electrochemical performance at ultrahigh charging/discharging conditions. This improvement is owing to the successful introduction of Mo doping and oxygen vacancies on the binder-free Co-CoO–based integrated electrode, which are beneficial for enhancing the electronic conductivity of CoO [46]. Figure 8b demonstrates that the Mo@Co-CoO IPMSCs3 microdevice exhibits good capacitive behavior at a super-high scan rate of up to 100 V s^−1^, which is around 500–1000 times higher than that of reported CoO-based electrodes. These include Cu-doped CoO electrodes prepared by combining the process of solvothermal and calcination [46], CoO-CNT–based electrodes prepared via the hydrothermal method [37], CoO nanoparticle-based electrodes fabricated through ball milling [73], and porous CoO nanowall-based electrodes manufactured by combining the techniques of solvothermal and annealing [74]. The high performance of the Mo@Co-CoO IPMSCs3 microdevice was realized without using any help from high-cost conductive additives (such as CNT and graphene), noble current collectors (like gold and silver), toxic chemicals, specific synthesis environments, and low-conductivity binders. Moreover, the Mo@Co-CoO IPMSCs3 microdevice was manufactured using EDM through a one-step process, greatly simplifying the fabrication procedures for IPMSCs. Figure 8c further proves that the Mo@Co-CoO IPMSCs3 obtained the maximum areal capacitance among the three microdevices—IPMSCs1, IPMSCs2, and IPMSCs3—at all super-high applied scan rates of 10, 20, 30, and 100 V s^−1^. This improvement is attributed to the reduction in electrode width and the 3D electrode design, accelerating the ion transport efficiency.

**Figure 9:**
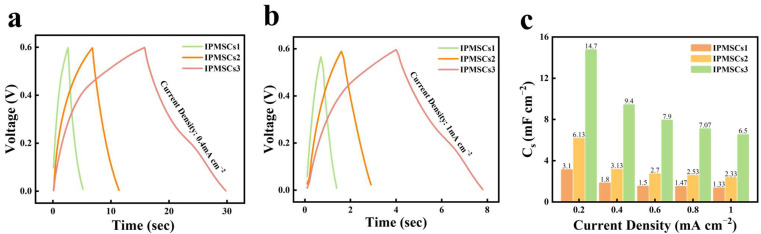
GCD profiles of IPMSCs1, IPMSCs2, and IPMSCs3 at current densities of (**a**) 0.4 mA cm^−2^ and (**b**) 1 mA cm^−2^, respectively, and (**c**) the related areal capacitance of IPMSCs1, IPMSCs2, and IPMSCs3.

Figure 9a,b show the GCD curves of IPMSCs1, IPMSCs2, and IPMSCs3 at the applied current densities of 0.4 mA cm^−2^ and 1 mA cm^−2^, respectively. The symmetric GCD curves of IPMSCs1, IPMSCs2, and IPMSCs3 imply good capacitive behavior for all the microdevices fabricated using the EDM technique. Compared with IPMSCs1 and IPMSCs2, IPMSCs3 displays a longer discharge time, indicating that IPMSCs3 obtains better capacitive performance. This improvement is attributed to the 3D microelectrode design, reducing the ion transport distance due to its smaller electrode width. This phenomenon is in good agreement with the results presented in Figure 7a. The related areal capacitances of IPMSCs1, IPMSCs2, and IPMSCs3, gained from GCD profiles, are shown in Figure 9c. It can be observed that the IPMSCs3 microdevices exhibited the maximum areal capacitance, and it gained 14.7 mF cm^−2^ at 0.2 mA cm^−2^ and 6.5 mF cm^−2^ at 1 mA cm^−2^. In comparison, the IPMSCs1 microdevice presented the minimum areal capacitance, and it gained 3.1 mF cm^−2^ at 0.2 mA cm^−2^ and 1.33 mF cm^−2^ at 1 mA cm^−2^. These results are consistent with those presented in Figure 7c and Figure 8c. Moreover, a good rate performance of 44.2% was achieved for IPMSCs3 when the current density was increased from 0.2 mA cm^−2^ to 1 mA cm^−2^. This improvement is because of the 3D Mo@Co-CoO–integrated microelectrode design, the absence of low-conductivity binders, and the introduction of Mo dopants and oxygen vacancies, facilitating fast electron transportation through the interface between the active materials of CoO and the current Co collector, while also boosting the ion transportation efficiency. Moreover, it needs to be noted that this good electrochemical performance was gained for the Mo@Co-CoO IPMSCs3 microdevices without using any help from high-cost conductive additives (such as CNT and graphene) and noble current collectors (like gold and silver). The whole manufacturing process is free from toxic chemicals, specific synthesis environments, and low-conductivity binders. This approach opens a new avenue for simplifying the synthesized procedures for materials, providing a novel way for realizing intelligent, digital, and green manufacturing of various metal oxide materials, microelectrode arrays, and microdevices.

## 4. Conclusions

In conclusion, a new manufacturing method using EDM, which is simple, safe, efficient, and environment-friendly, has been developed for the one-step fabrication of Mo@Co-CoO–integrated microelectrodes with Mo doping and enriched with oxygen vacancies for efficiently constructing Mo@Co-CoO IPMSCs with customized geometric shape and tailorable electrochemical performance. The results demonstrate that the Mo@Co-CoO IPMSCs3, with the smallest electrode width, gained a maximum areal capacitance of 30.4 mF cm^−2^ at 2 mV s^−1^. In contrast, the Mo@Co-CoO IPMSCs1, with the largest electrode width, exhibited the minimum capacitance. This difference is owing to the reduction in electrode width, boosting the ion transportation efficiency. Moreover, the Mo@Co-CoO IPMSCs3 showed good rate performance and capacitive behavior at a super-high scan rate of 100 V s^−1^, which is around 500–1000 times higher than those of reported CoO-based electrodes. This improvement is because of the 3D Mo@Co-CoO–integrated microelectrode design, the absence of low-conductivity binders, and the introduction of Mo dopants and oxygen vacancies, which facilitate fast electron transportation through the interface between the active CoO materials and the current Co collector, further boosting ion transportation efficiency. It is important to note that all the IPMSCs were fabricated using one-step EDM without any assistance of other materials processing techniques, toxic chemicals, low-conductivity binders, exceptional current collectors, and conductive fillers. This fabrication method, developed in this research, opens a new avenue for simplifying the preparation of materials and microdevices, providing a novel way for realizing intelligent, digital, and green manufacturing of various metal oxide materials, microelectrode arrays, and microdevices.

## Data Availability

The original contributions presented in the work are included in the article, further inquiries can be directed to the corresponding author.

## Supplementary Materials

The following supporting information can be downloaded at https://www.mdpi.com/article/10.3390/mi15111294/s1.

## References

[1] Lee S.H., Lee J., Jung J., Cho A.R., Jeong J.R., Dang Van C., Nah J., Lee M.H. (2021). Enhanced electrochemical performance of micro-supercapacitors via laser-scribed cobalt/reduced graphene oxide hybrids. ACS Appl. Mater. Interfaces.

[2] Zhu Y., Zhang Q., Ma J., Das P., Zhang L., Liu H., Wang S., Li H., Wu Z.S. (2024). Three-dimensional (3D)-printed MXene high-voltage aqueous micro-supercapacitors with ultrahigh areal energy density and low-temperature tolerance. Carbon Energy.

[3] Zhu Y., Wang S., Ma J., Das P., Zheng S., Wu Z.-S. (2022). Recent status and future perspectives of 2D MXene for micro-supercapacitors and micro-batteries. Energy Storage Mater..

[4] Jia J., Zhu Y., Das P., Ma J., Wang S., Zhu G., Wu Z.-S. (2023). Advancing MXene-based integrated microsystems with micro-supercapacitors and/or sensors: Rational design, key progress, and challenging perspectives. J. Mater..

[5] Li L., Hu C., Liu W., Shen G. (2021). Progress and perspectives in designing flexible microsupercapacitors. Micromachines.

[6] Dinh K.H., Roussel P., Lethien C. (2023). Advances on microsupercapacitors: Real fast miniaturized devices toward technological dreams for powering embedded electronics?. ACS Omega.

[7] Atzori L., Iera A., Morabito G. (2010). The internet of things: A survey. Comput. Netw..

[8] Khodabandehlo A., Noori A., Rahmanifar M.S., El-Kady M.F., Kaner R.B., Mousavi M.F. (2022). Laser-Scribed Graphene–Polyaniline Microsupercapacitor for Internet-of-Things Applications. Adv. Funct. Mater..

[9] Gao C., Huang J., Xiao Y., Zhang G., Dai C., Li Z., Zhao Y., Jiang L., Qu L. (2021). A seamlessly integrated device of micro-supercapacitor and wireless charging with ultrahigh energy density and capacitance. Nat. Commun..

[10] Hou R., Gund G.S., Qi K., Nakhanivej P., Liu H., Li F., Xia B.Y., Park H.S. (2019). Hybridization design of materials and devices for flexible electrochemical energy storage. Energy Storage Mater..

[11] Shen D., Xiao M., Zou G., Liu L., Duley W.W., Zhou Y.N. (2018). Wearable Electronics: Self-Powered Wearable Electronics Based on Moisture Enabled Electricity Generation (Adv. Mater. 18/2018). Adv. Mater..

[12] Zhang X.-S., Han M., Kim B., Bao J.-F., Brugger J., Zhang H. (2018). All-in-one self-powered flexible microsystems based on triboelectric nanogenerators. Nano Energy.

[13] Wang J., Li F., Zhu F., Schmidt O.G. (2019). Recent progress in micro-supercapacitor design, integration, and functionalization. Small Methods.

[14] Haider W.A., Tahir M., He L., Mirza H., Zhu R., Han Y., Mai L. (2020). Structural engineering and coupling of two-dimensional transition metal compounds for micro-supercapacitor electrodes. ACS Cent. Sci..

[15] Kyeremateng N.A., Brousse T., Pech D. (2017). Microsupercapacitors as miniaturized energy-storage components for on-chip electronics. Nat. Nanotechnol..

[16] Jang S., Kang J., Kwak S., Seol M.-L., Meyyappan M., Nam I. (2021). Methodologies for fabricating flexible supercapacitors. Micromachines.

[17] Chen R., Yu M., Sahu R.P., Puri I.K., Zhitomirsky I. (2020). The development of pseudocapacitor electrodes and devices with high active mass loading. Adv. Energy Mater..

[18] Li Y., Ni L., Luo J., Zhu L., Zhang X., Li H., Zada I., Yu J., Zhu S., Lian K. (2024). Fenton Reaction Doubled Biomass Carbon Activation Efficiency for High-Performance Supercapacitors. Adv. Funct. Mater..

[19] Wang Y., Sun L., Xiao D., Du H., Yang Z., Wang X., Tu L., Zhao C., Hu F., Lu B. (2020). Silicon-based 3D all-solid-state micro-supercapacitor with superior performance. ACS Appl. Mater. Interfaces.

[20] Shen C., Wang X., Zhang W., Kang F. (2011). A high-performance three-dimensional micro supercapacitor based on self-supporting composite materials. J. Power Sources.

[21] Tian W., Li Y., Zhou J., Wang T., Zhang R., Cao J., Luo M., Li N., Zhang N., Gong H. (2021). Implantable and biodegradable micro-supercapacitor based on a superassembled three-dimensional network Zn@ PPy hybrid electrode. ACS Appl. Mater. Interfaces.

[22] Azadmanjiri J., Reddy T.N., Khezri B., Děkanovský L., Parameswaran A.K., Pal B., Ashtiani S., Wei S., Sofer Z. (2022). Prospective advances in MXene inks: Screen printable sediments for flexible micro-supercapacitor applications. J. Mater. Chem. A.

[23] Yang M., Kasbe P., Bu J., Xu W. (2024). Scalable solid-state synthesis of 2D transition metal oxide/graphene hybrid materials and their utilization for microsupercapacitors. Nanoscale.

[24] Mohanty R.I., Mukherjee A., Bhanja P., Jena B.K. (2023). Novel microporous manganese phosphonate-derived metal oxides as prospective cathode materials for superior flexible asymmetric micro-supercapacitor device. J. Energy Storage.

[25] Liu W., Zhang Z., Zhang Y., Zheng Y., Liu N., Su J., Gao Y. (2021). Interior and exterior decoration of transition metal oxide through Cu^0^/Cu^+^ Co-doping strategy for high-performance supercapacitor. Nano-Micro Lett..

[26] Asbani B., Buvat G., Freixas J., Huvé M., Troadec D., Roussel P., Brousse T., Lethien C. (2021). Ultra-high areal capacitance and high rate capability RuO_2_ thin film electrodes for 3D micro-supercapacitors. Energy Storage Mater..

[27] Chang Y., Li P., Li L., Chang S., Huo Y., Mu C., Nie A., Xiang J., Xue T., Zhai K. (2021). In situ grown ultrafine RuO_2_ nanoparticles on GeP5 nanosheets as the electrode material for flexible planar micro-supercapacitors with high specific capacitance and cyclability. ACS Appl. Mater. Interfaces.

[28] Seenath J.S., Pech D., Rochefort D. (2022). Investigation of protic ionic liquid electrolytes for porous RuO_2_ micro-supercapacitors. J. Power Sources.

[29] Bounor B., Asbani B., Douard C., Favier F., Brousse T., Lethien C. (2021). On chip MnO_2_-based 3D micro-supercapacitors with ultra-high areal energy density. Energy Storage Mater..

[30] Chen R., Poon R., Sahu R.P., Puri I.K., Zhitomirsky I. (2017). MnO_2_-carbon nanotube electrodes for supercapacitors with high active mass loadings. J. Electrochem. Soc..

[31] Xu Y., Deng P., Chen R., Xie W., Xu Z., Yang Y., Liu D., Huang F., Zhuang Z., Zhitomirsky I. (2023). Electric discharge direct writing of 3D Mo-MoOx pseudocapacitive micro-supercapacitors with designable patterns. Ceram. Int..

[32] Chen R., Qin J., Xu Z., Lv S., Tao Z., He J., Zhou P., Shu Z., Zhuang Z., Wang W. (2024). The impact of processing voltage of wire electric discharge machining on the performance of Mo doped V–VO_0.2_ based Archimedean micro-supercapacitors. RSC Adv..

[33] Zheng Y., Liu H., Chen Y., Hou M., Gao J., Chen X. In situ depositing Fe_3_O_4_ nanoparticles on laser-induced graphene for high performance microsupercapacitors. Proceedings of the 2022 23rd International Conference on Electronic Packaging Technology (ICEPT).

[34] Liu D., Xie W., Xu Z., Deng P., Wu Z., Zhitomirsky I., Wang W., Chen R., Zhou L., Xu Y. (2023). Fabrication of Ni-Cr-FeOx ceramic supercapacitor electrodes and devices by one-step electric discharge ablation. J. Energy Storage.

[35] Chen R., Xu Z., Xie W., Deng P., Xu Y., Xu L., Zhang G., Yang Y., Xie G., Zhitomirsky I. (2023). Fabrication of Fe–Fe_1−x_ O based 3D coplanar microsupercapacitors by electric discharge rusting of pure iron substrates. RSC Adv..

[36] Nawwar M., Poon R., Chen R., Sahu R.P., Puri I.K., Zhitomirsky I. (2019). High areal capacitance of Fe_3_O_4_-decorated carbon nanotubes for supercapacitor electrodes. Carbon Energy.

[37] Zhu Y.G., Wang Y., Shi Y., Wong J.I., Yang H.Y. (2014). CoO nanoflowers woven by CNT network for high energy density flexible micro-supercapacitor. Nano Energy.

[38] Sun G., Yang H., Zhang G., Gao J., Jin X., Zhao Y., Jiang L., Qu L. (2018). A capacity recoverable zinc-ion micro-supercapacitor. Energy Environ. Sci..

[39] Zheng D., Zhao F., Li Y., Qin C., Zhu J., Hu Q., Wang Z., Inoue A. (2019). Flexible NiO micro-rods/nanoporous Ni/metallic glass electrode with sandwich structure for high performance supercapacitors. Electrochim. Acta.

[40] Abdalla A.M., Sahu R.P., Wallar C.J., Chen R., Zhitomirsky I., Puri I.K. (2017). Nickel oxide nanotube synthesis using multiwalled carbon nanotubes as sacrificial templates for supercapacitor application. Nanotechnology.

[41] Wang L., Ding Y., Li J., Guan Y., Yang L., Fang H., Lv X., Yuan J. (2022). Femtosecond laser induced one-step nanopatterning and preparation of rGO/RuO_2_ electrodes for high-performance micro-supercapacitors. J. Electroanal. Chem..

[42] Karimi F., Korkmaz S., Karaman C., Karaman O., Kariper İ.A. (2022). Engineering of GO/MWCNT/RuO_2_ ternary aerogel for high-performance supercapacitor. Fuel.

[43] Shewale P.S., Yun K.-S. (2024). Editorial for the Special Issue on Graphene-Nanocomposite-Based Flexible Supercapacitors. Micromachines.

[44] Zhou C., Zhang Y., Li Y., Liu J. (2013). Construction of high-capacitance 3D CoO@ polypyrrole nanowire array electrode for aqueous asymmetric supercapacitor. Nano Lett..

[45] Gao M., Dong X., Mei X., Wang K., Wang W., Tang Z., Duan W. (2023). Laser direct synthesis of Co/CoO modified graphene for high-performance microsupercapacitors. Chem. Eng. J..

[46] Feng Y., Sun L., Qi Z., Zhang Y., Wang G., Gao W., Liu W. (2023). Cationic and anionic defect decoration of CoO through Cu dopants and oxygen vacancy for a High-Performance supercapacitor. J. Colloid Interface Sci..

[47] Muthuramalingam T., Mohan B. (2015). A review on influence of electrical process parameters in EDM process. Arch. Civ. Mech. Eng..

[48] Popa M.S., Contiu G., Pop G., Dan P. (2009). New technologies and applications of EDM process. Int. J. Mater. Form..

[49] Qu J., Riester L., Shih A.J., Scattergood R.O., Lara-Curzio E., Watkins T.R. (2003). Nanoindentation characterization of surface layers of electrical discharge machined WC–Co. Mater. Sci. Eng. A.

[50] Liang P., Zhang H., Pan B., Su Y., Wang C.A., Zhong M. (2019). Binder-free carbon-coated nanocotton transition metal oxides integrated anodes by laser surface ablation for lithium-ion batteries. Surf. Interface Anal..

[51] Xia P., Pan J., Zhang Y., Mao M., Ma L., Chen J., Zhang L., Wang H., Fan H., Gao X. (2024). Highly sensitive detection of glucose at a novel non-enzyme electrochemical sensing based on Mo-doped CoO Nanosheets. Chem.–Asian J..

[52] Song T., Qi Y., Jia A., Ta N., Lu J., Wu P., Li X. (2021). Continuous hydrogenation of CO_2_-derived ethylene carbonate to methanol and ethylene glycol at Cu-MoOx interface with a low H_2_/ester ratio. J. Catal..

[53] Yuan H., Wang S., Ma Z., Kundu M., Tang B., Li J., Wang X. (2021). Oxygen vacancies engineered self-supported B doped Co_3_O_4_ nanowires as an efficient multifunctional catalyst for electrochemical water splitting and hydrolysis of sodium borohydride. Chem. Eng. J..

[54] Liu S., Yang Y., Zhong M., Li S., Shi S., Xiao W., Wang S., Chen C. (2023). Constructing an efficient electrocatalyst for water oxidation: An Fe-doped CoO/Co catalyst enabled by in situ MOF growth and a solvent-free strategy. Dalton Trans..

[55] Mestl G., Srinivasan T. (1998). Raman spectroscopy of monolayer-type catalysts: Supported molybdenum oxides. Catal. Rev..

[56] Kumar R., Sahoo S., Joanni E., Pandey R., Tan W.K., Kawamura G., Moshkalev S.A., Matsuda A. (2023). Microwave-assisted dry synthesis of hybrid electrode materials for supercapacitors: Nitrogen-doped rGO with homogeneously dispersed CoO nanocrystals. J. Energy Storage.

[57] Jiang Z., Yang H., Cao L., Yang Z., Yuan Y., Li E. (2021). Enhanced breakdown strength and energy storage density of lead-free Bi0. 5Na0.5TiO_3_-based ceramic by reducing the oxygen vacancy concentration. Chem. Eng. J..

[58] Gao D., Zhang J., Yang G., Qi J., Si M., Xue D. (2011). Ferromagnetism induced by oxygen vacancies in zinc peroxide nanoparticles. J. Phys. Chem. C.

[59] Han Y., Wu S., Dai E., Ye Y., Liu J., Tian Z., Cai Y., Zhu X., Liang C. (2017). Laser-Irradiation-Induced Melting and Reduction Reaction for the Formation of Pt-Based Bimetallic Alloy Particles in Liquids. ChemPhysChem.

[60] Sundriyal P., Bhattacharya S. (2019). Scalable micro-fabrication of flexible, solid-state, inexpensive, and high-performance planar micro-supercapacitors through inkjet printing. ACS Appl. Energy Mater..

[61] Wu Z.-S., Parvez K., Feng X., Müllen K. (2014). Photolithographic fabrication of high-performance all-solid-state graphene-based planar micro-supercapacitors with different interdigital fingers. J. Mater. Chem. A.

[62] Wang K., Wu H., Zou W., Quan B., Yu A., Jiang P., Wei Z. (2011). An all-solid-state flexible micro-supercapacitor on a chip. Adv. Energy Mater..

[63] Dewan A., Narayanan R., Thotiyl M.O. (2022). A multi-chromic supercapacitor of high coloration efficiency integrating a MOF-derived V_2_O_5_ electrode. Nanoscale.

[64] Boruah B.D., Nandi S., Misra A. (2018). Layered assembly of reduced graphene oxide and vanadium oxide heterostructure supercapacitor electrodes with larger surface area for efficient energy-storage performance. ACS Appl. Energy Mater..

[65] Liu Z., Wu Z.-S., Yang S., Dong R., Feng X., Müllen K. (2016). Ultraflexible in-plane micro-supercapacitors by direct printing of solution-processable electrochemically exfoliated graphene. Adv. Mater..

[66] Jiang Q., Wu C., Wang Z., Wang A.C., He J.-H., Wang Z.L., Alshareef H.N. (2018). MXene electrochemical microsupercapacitor integrated with triboelectric nanogenerator as a wearable self-charging power unit. Nano Energy.

[67] Yang Y., He L., Tang C., Hu P., Hong X., Yan M., Dong Y., Tian X., Wei Q., Mai L. (2016). Improved conductivity and capacitance of interdigital carbon microelectrodes through integration with carbon nanotubes for micro-supercapacitors. Nano Res..

[68] Pech D., Brunet M., Taberna P.-L., Simon P., Fabre N., Mesnilgrente F., Conédéra V., Durou H. (2010). Elaboration of a microstructured inkjet-printed carbon electrochemical capacitor. J. Power Sources.

[69] Pech D., Brunet M., Durou H., Huang P., Mochalin V., Gogotsi Y., Taberna P.-L., Simon P. (2010). Ultrahigh-power micrometre-sized supercapacitors based on onion-like carbon. Nat. Nanotechnol..

[70] Wu Z., Parvez K., Feng X., Müllen K. (2013). Graphene-based in-plane micro supercapacitors with high power and energy densities. Nat. Commun..

[71] El-Kady M.F., Kaner R.B. (2013). Scalable fabrication of high-power graphene micro-supercapacitors for flexible and on-chip energy storage. Nat. Commun..

[72] Gao W., Singh N., Song L., Liu Z., Reddy A.L.M., Ci L., Vajtai R., Zhang Q., Wei B., Ajayan P.M. (2011). Direct laser writing of micro-supercapacitors on hydrated graphite oxide films. Nat. Nanotechnol..

[73] Zheng C., Cao C., Ali Z., Hou J. (2014). Enhanced electrochemical performance of ball milled CoO for supercapacitor applications. J. Mater. Chem. A.

[74] Tang N., Wang W., You H., Zhai Z., Hilario J., Zeng L., Zhang L. (2019). Morphology tuning of porous CoO nanowall towards enhanced electrochemical performance as supercapacitors electrodes. Catal. Today.

