# Supplementary material for: 3D Binder-Free Mo@CoO Electrodes Directly Manufactured in One Step via Electric Discharge Machining for In-Plane Microsupercapacitor Application
Source: Micromachines (Basel). 2024 Oct 24;15(11):1294. doi: 10.3390/mi15111294 (PMC11596131; doi:10.3390/mi15111294)
Supplement: Supplementary file 1 [file micromachines-15-01294-s001.zip › micromachines-3249994-supplementary.pdf]

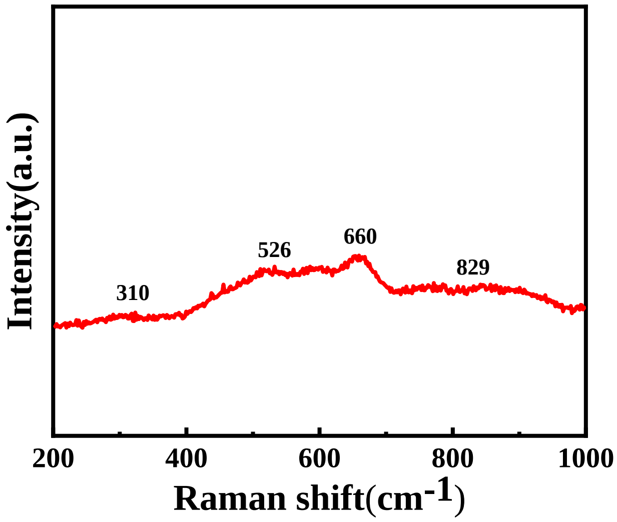

Figure S1. Raman profile of the Mo@Co-CoO integrated electrode.

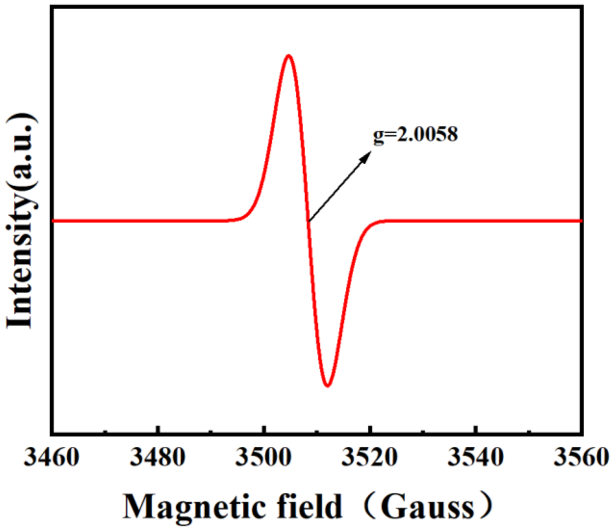

Figure S2. EPR profile of the Mo@Co-CoO integrated electrode.

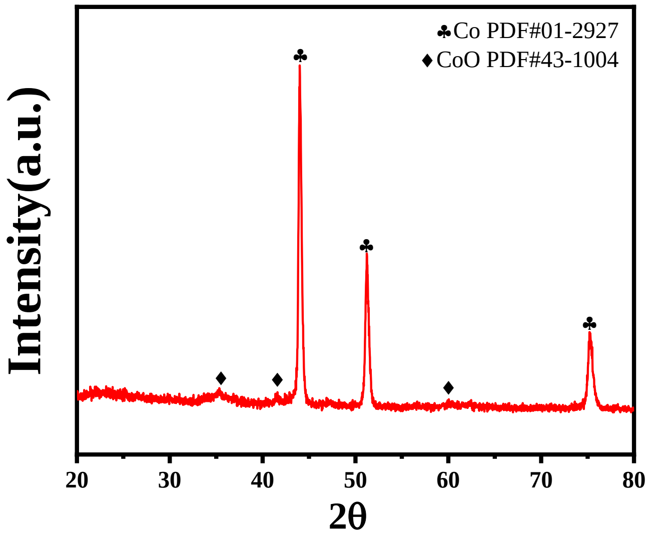

Figure S3. XRD pattern of the Mo@Co-CoO integrated electrode.

Table S1. Areal capacitance of MSCs fabricated by various techniques.

Devices	Fabricated Techniques	Current collectors	Capacitance (mF cm^{-2})	References
V_2O_5//PANI	Electrodeposition Spray coating	FTO	12.3	[1]
V_2O_5//rGO	Spray coating, Sputtering	Au/Cr current collectors	24	[2]
Graphene–PEDOT	Mask–assisted spray deposition, Electrochemically exfoliating	Integrated electrodes	5.4	[3]
MXene	Laser machining Spray–coating	Integrated electrodes	23	[4]
CNT	Spin coating, Photolithography	Integrated electrodes	6.1	[5]
Activated carbon	Inkjet printing, Photolithography, Chemical vapour deposition	Gold current collector	5.1	[6]
Carbon onions	Electrophoretic deposition, Photolithography	Gold current collector	1.7	[7]
Graphene	Spin coating, Lithography	Gold current collector	0.08	[8]
Graphene	Laser–scribing	Integrated electrodes	2.3	[9]
rGO	Laser radiation, Vacuum filtration	Carbon–coated aluminium foils	0.51	[10]
Mo@Co-CoO IPMSCs3	NCEDM	Integrated electrodes	30.4	**This work**

References

[1] A. Dewan, R. Narayanan, M. O. Thotiyl, A multi–chromic supercapacitor of high coloration efficiency integrating a MOF–derived V_2O_5 electrode, Nanoscale 14(46) (2022) 17372–17384.

[2] B. D. Boruah, S. Nandi, A. Misra, Layered assembly of reduced graphene oxide and vanadium oxide heterostructure supercapacitor electrodes with larger surface area for efficient energy–storage performance, ACS Applied Energy Materials 1(4) (2018)

1567–1574.
[3] Z. Liu, Z. S. Wu, S. Yang, R. Dong, X. Feng, K. Müllen, Ultraflexible in–plane micro–supercapacitors by direct printing of solution–processable electrochemically exfoliated graphene, Advanced Materials 28(11) (2016) 2217–2222.
[4] Q. Jiang, C. Wu, Z. Wang, A. C. Wang, J. H. He, Z. L. Wang, H. N. Alshareef, MXene electrochemical micro–supercapacitor integrated with triboelectric nanogenerator as a wearable self–charging power unit, Nano Energy 45 (2018) 266–272.
[5] Y. Yang, L. He, C. Tang, P. Hu, X. Hong, M. Yan, Y. Dong, X. Tian, Q. Wei, L. Mai, Improved conductivity and capacitance of interdigital carbon microelectrodes through integration with carbon nanotubes for micro–supercapacitors, Nano Research 9 (2016) 2510–2519.
[6] D. Pech, M. Brunet, P. L. Taberna, P. Simon, N. Fabre, F. Mesnilgrente, V. Conédéra, H. Durou, Elaboration of a microstructured inkjet–printed carbon electrochemical capacitor, Journal of Power Sources 195(4) (2010) 1266–1269.
[7] D. Pech, M. Brunet, H. Durou, P. Huang, V. Mochalin, Y. Gogotsi, P. L. Taberna, P. Simon, Ultrahigh–power micrometre–sized supercapacitors based on onion–like carbon, Nature nanotechnology 5(9) (2010) 651–654.
[8] Z. S. Wu, K. Parvez, X. Feng, K. Müllen, Graphene–based in–plane micro–supercapacitors with high power and energy densities, Nature communications 4(1) (2013) 2487.
[9] M. F. El–Kady, R. B. Kaner, Scalable fabrication of high–power graphene micro–supercapacitors for flexible and on–chip energy storage, Nature communications 4(1) (2013) 1475.
[10] W. Gao, N. Singh, L. Song, Z. Liu, A. L. M. Reddy, L. Ci, R. Vajtai, Q. Zhang, B. Wei, P.M. Ajayan, Direct laser writing of micro–supercapacitors on hydrated graphite oxide films, Nature nanotechnology 6(8) (2011) 496–500.
